# Combination anti‐HIV antibodies to achieve antiretroviral therapy‐free virological suppression in infected individuals

**DOI:** 10.1002/ctm2.1057

**Published:** 2022-09-13

**Authors:** M. Ali Rai, Tae‐Wook Chun

**Affiliations:** ^1^ HIV Immunovirology Section Laboratory of Immunoregulation National Institute of Allergy and Infectious Diseases National Institutes of Health Bethesda Maryland USA

1

Over the past 26 years, the discovery and widespread implementation of antiretroviral therapy (ART) has led to dramatic improvements in the management of HIV diseases and a marked reduction of HIV‐related morbidity and mortality.^1^ The vast majority of HIV‐infected individuals who are adherent to ART can suppress their plasma viremia.^2^ However, ART is not curative and HIV‐infected individuals must continue life‐long therapy to maintain virologic suppression.^3^, ^4^ To mitigate the complications associated with long‐term ART (adherence, side effects, resistance, and economic considerations), significant efforts have been made to find effective alternatives that could lead to ART‐free virologic suppression in infected individuals.^5^ However, despite more than a decade of intense research, the prospects for finding a cure for HIV remain elusive, if not impossible.^6^ Rather, an approach involving intermittent administration of long‐acting therapeutic agents, which may not necessarily eliminate the pre‐existing infected cells, has emerged as an alternative to achieve virologic suppression in infected individuals.^3^ In this regard, long‐acting injectable antiretroviral drugs hold promise for suppressing plasma viremia with infrequent dosing.^7^, ^8^, ^9^ In addition, broadly neutralizing monoclonal antibodies (bNAbs) against HIV have emerged as a promising therapeutic modality to achieve durable suppression of plasma viremia for extended periods.^10^, ^11^

The first group of monoclonal antibodies against HIV exhibiting in vitro neutralization activity were described in the early 1990s.^12^ Although some of these antibodies showed promising results, subsequent studies in humans generated less favorable outcomes.^12^, ^13^ Since then, rapid technological advancement, particularly with the advent of single‐cell antibody cloning and sequencing techniques, has led to the isolation and clinical development of bNAbs with increased breadth and therapeutic efficacy.^11^ The first human trials involving passive transfer of single bNAbs, VRC01 and 3BNC117 that target the CD4 binding site on the HIV Env protein, were conducted between 2015–2017 in infected individuals undergoing analytical treatment interruption (ATI).^14^, ^15^ Although these studies demonstrated a moderate delay in plasma viral rebound compared to historical data, the pre‐existing and emergent bNAb‐resistant HIV abrogated the prospect of long‐term control in study participants.^14^, ^15^ A follow‐up study involving three infusions of two bNAbs 3BNC117 and 10–1074 (specificity to V3 loop and glycans on HIV Env) over the period of 6 weeks demonstrated a significant delay in plasma viral rebound in infected individuals following ATI.^16^ These encouraging results paved the way to follow‐up clinical trials aimed at addressing the long‐term impact of the combination of these bNAbs on virologic suppression, viral reservoirs, and immune cells in HIV‐infected individuals.

To this end, two research groups recently conducted clinical trials to address the feasibility of achieving long‐term (>6 months) virologic suppression in HIV‐infected individuals following ATI.^14^, ^17^ Gaebler et al.^17^ conducted a phase 1b, open‐label, clinical trial involving 23 HIV‐infected study participants. Among them, 17 study participants underwent ATI shortly after receiving the first round of infusions of 3BNC117 (30 mg/kg) and 10–1074 (30 mg/kg). Thirteen study participants (76%) achieved virologic suppression for at least 20 weeks with a median time of 28.5 weeks to plasma viral rebound (>200 copies/ml). For those who achieved long‐term virologic suppression during ATI, their plasma viremia eventually rebounded when serum concentrations of one of the bNAbs fell below the therapeutic level (10μg/ml). Remarkably, Gaebler et al. observed modest but statistically significant reduction in the size of HIV reservoirs carrying the intact, but not defective proviral DNA, in the CD4^+^ T cell compartment in whom plasma viremia was suppressed following passive transfer of the two bNAbs.^17^ In a separate study, Sneller et al. conducted a randomized, double‐blind, placebo‐controlled trial that enrolled participants who initiated ART during the acute/early phase of HIV infection.^18^ The participants in the bNAb arm received up to eight infusions of 3BNC117 (30 mg/kg) and 10–1074 (30 mg/kg) over a period of 24 weeks. The pre‐defined virologic endpoint of this study was the difference between the bNAb and placebo arms in the number of participants who experienced viral rebound and met ART restart criteria before reaching study week 28. Sneller et al. demonstrated that none of the study participants in the bNAb arm restarted ART prior to study week 28 (median 39.6 weeks), whereas 86% of the participants in the placebo arm did.^18^ The study showed no evidence for the diminution of the persistent HIV reservoir in the participants in the bNAb arm who achieved sustained virologic suppression nor the difference between the two arms in the level of plasma viremia prior to re‐initiation of ART.

Contrary to the existing conventional or long‐acting injectable ART regimens, bNAbs may offer additional and unique advantages, such as accelerated clearance of the persistent HIV reservoirs^19^ and enhance antiviral immunity in infected individuals.^20^, ^21^ In this regard, a previous study has demonstrated increased levels of HIV Gag‐specific CD8^+^ T cells in study participants who had received infusions of 3BNC117 and 10–1074.^22^ Nonetheless, further studies involving an extensive evaluation of cytotoxic CD8^+^ T cells as well as other immune cells will be necessary to better understand how bNAbs modulate cell‐mediated antiviral activities in vivo.

One of the major disadvantages of bNAbs as a therapeutic option for the treatment of HIV over conventional antiretroviral drugs is that a substantial proportion of infected individuals carry antibody‐resistant, pre‐existing viral reservoirs.^14^, ^15^ The two aforementioned long‐term studies did not screen study participants for the presence of 3BNC117‐ and/or 10‐1074‐resistant virus prior to administration of the antibodies and clearly demonstrated that those with resistance to one of the two bNAbs failed to suppress their plasma viremia for prolonged periods of time.^17^, ^18^ To maximize long‐term therapeutic potential, it would be necessary to develop reliable, high throughput and clinically relevant screening methodologies to identify individuals with antibody‐resistant, pre‐existing HIV reservoirs prior to study enrollment. Next generation bNAbs with increased breadth and antiviral efficacy, extended half‐lives and possibly with a long‐acting injectable antiretroviral drugs administered every 6 weeks could lead to prolonged virologic suppression in infected individuals without the burden of taking daily ART.

## CONFLICT OF INTEREST

The authors have declared no conflict of interest exists.
